# Berberine Decreases Intestinal GLUT2 Translocation and Reduces Intestinal Glucose Absorption in Mice

**DOI:** 10.3390/ijms23010327

**Published:** 2021-12-28

**Authors:** Min Zhang, Hongyan Yang, Erwan Yang, Jia Li, Ling Dong

**Affiliations:** Key Laboratory of Aerospace Medicine of the Ministry of Education, School of Aerospace Medicine, Air Force Military Medical University, Xi’an 710032, China; zhangmin2015@fmmu.edu.cn (M.Z.); yhy2013@fmmu.edu.cn (H.Y.); yang1123665336@sina.com (E.Y.); jiali816@fmmu.edu.cn (J.L.)

**Keywords:** berberine, intestinal epithelial cells, glucose absorption, GLUT2

## Abstract

Postprandial hyperglycemia is an important causative factor of type 2 diabetes mellitus, and permanent localization of intestinal GLUT2 in the brush border membrane is an important reason of postprandial hyperglycemia. Berberine, a small molecule derived from Coptidis rhizome, has been found to be potent at lowering blood glucose, but how berberine lowers postprandial blood glucose is still elusive. Here, we investigated the effect of berberine on intestinal glucose transporter 2 (GLUT2) translocation and intestinal glucose absorption in type 2 diabetes mouse model. Type 2 diabetes was induced by feeding of a high-fat diet and injection of streptozotocin and diabetic mice were treated with berberine for 6 weeks. The effects of berberine on intestinal glucose transport and GLUT2 translocation were accessed in isolated intestines and intestinal epithelial cells (IEC-6), respectively. We found that berberine treatment improved glucose tolerance and systemic insulin sensitivity in diabetic mice. Furthermore, berberine decreased intestinal glucose transport and inhibited GLUT2 translocation from cytoplasm to brush border membrane in intestinal epithelial cells. Mechanistically, berberine inhibited intestinal insulin-like growth factor 1 (IGF-1R) phosphorylation and thus reduced localization of PLC-β2 in the membrane, leading to decreased GLUT2 translocation. These results suggest that berberine reduces intestinal glucose absorption through inhibiting IGF-1R-PLC-β2-GLUT2 signal pathway.

## 1. Introduction

The absorption of nutrients is the primary function of the small intestine, and abnormal intestinal absorption often leads to various metabolic diseases. As for type 2 diabetes mellitus (T2DM), pathological enhancement of intestinal glucose absorption has been considered as one of the major causal factors of postprandial hyperglycemia, which is closely related to complications and prognosis of T2DM [1]. However, there are hardly any highly recommended medications to rescue pathological changes of intestinal glucose absorption directly in T2DM management.

Intestinal glucose absorption mainly depends on sodium-glucose cotransporter 1 (SGLT1) and glucose transporter 2 (GLUT2) in epithelial cells [2]. Luminal glucose is transported into epithelial cells through SGLT1 in the brush border membrane (BBM) and transported from intestinal epithelial cells to mesenteric vein through GLUT2 in the basolateral membrane (BLM) when the luminal concentration of glucose is lower than 30 mmol/L. However, with higher concentration of luminal glucose in postprandial condition, cytoplastic GLUT2 is translocated to BBM rapidly and transports glucose by facilitated diffusion, and thus, GLUT2-mediated glucose diffusion becomes the dominant approach of glucose absorption after meals [3]. Insulin and other hormones that promote secretion of insulin are upregulated; with increased blood glucose, insulin acts as a limiter of intestinal glucose absorption through internalizing GLUT2 from BBM to cytoplasm [4]. The translocation and internalization of GLUT2 remain a dynamic balance in healthy individuals and contribute to glucose homeostasis. However, with this dynamic balance disturbed in T2DM, the permanent localization of GLUT2 in BBM is closely related to the pathological enhancement of glucose absorption and postprandial hyperglycemia [5]. There are so far few medicines to rectify abnormal intestinal GLUT2 translocation.

Various mechanisms are involved in the regulation of intracellular translocation of GLUT2 in the intestine epithelial cells [6]. With increased concentration of luminal glucose over 20 mmol/L under postprandial condition, G-protein-mediated signaling systems are activated, which promote phospholipase C (PLC) β2 recruitment to the plasma membrane, thereby increasing intracellular Ca^2+^, which activates protein kinase C βII (PKCβII), then stimulates phosphorylation of myosin II and promotes the translocation of GLUT2 from cytoplasm to BBM [7,8]. Given the important role of PLC-β2, it is a worthy scientific question whether PLC-β2 can be a target to reduce GLUT2 translocation and improve postprandial hyperglycemia in T2DM.

Berberine, a small molecule derived from Coptidis rhizome, has been shown a potential as an adjunct treatment of T2DM [9]. Berberine’s anti-diabetic effects are mainly manifested as improving glycolipid metabolism, reducing risk factors and complications of diabetes [10]. After oral administration, berberine accumulates in multiple organs and tissues, such as the liver, muscle, adipose tissue, heart, and kidney [11], acting as a modulator of glucose and lipid homeostasis. Berberine increases glucose consumption and insulin sensitivity by activating AMPK, suppressing inflammatory responses and ROS. In addition, berberine lowers cholesterol level, inhibits lipogenesis, and induces energy expenditure [12]. Our preliminary study suggests that berberine reduces diabetic myocardial damage by inhibiting IGF-1R signal [13]. However, berberine absorption and the first-pass elimination mainly occurs in the small intestine, with over 99% of berberine not eventually entering the blood circulation [14]. Given the massive accumulation in the intestinal cavity, the intestinal mechanisms of berberine’s biological effects have been studied recently. It is concluded that berberine improves the structure of gastrointestinal microbiota and exerts indirect metabolic benefits by increasing short chain fatty acid [15,16]. It has also been found that berberine can reduce the activity of disaccharidase and inhibit the digestion of carbohydrates in the intestine [17]. Through G-protein-mediated signal pathway, berberine induces GLP-1 section in intestinal enteroendocrine cells [18]. However, it is still unclear whether berberine has a direct effect on G-protein-mediated glucose absorption in intestinal epithelial cells, especially in T2DM.

This study aims to determine the effects of berberine on the intestinal glucose absorption and postprandial hyperglycemia in T2DM. We found that berberine decreased GLUT2 localization in the BBM of intestinal epithelium through suppressingIGF-1R-PLC-β2-GLUT2 signaling.

## 2. Results

### 2.1. Berberine Alleviated Hyperglycemia, Improved Insulin Sensitivity and Reduced Intestinal Glucose Absorption in Diabetic Mice

**Figure 1:**
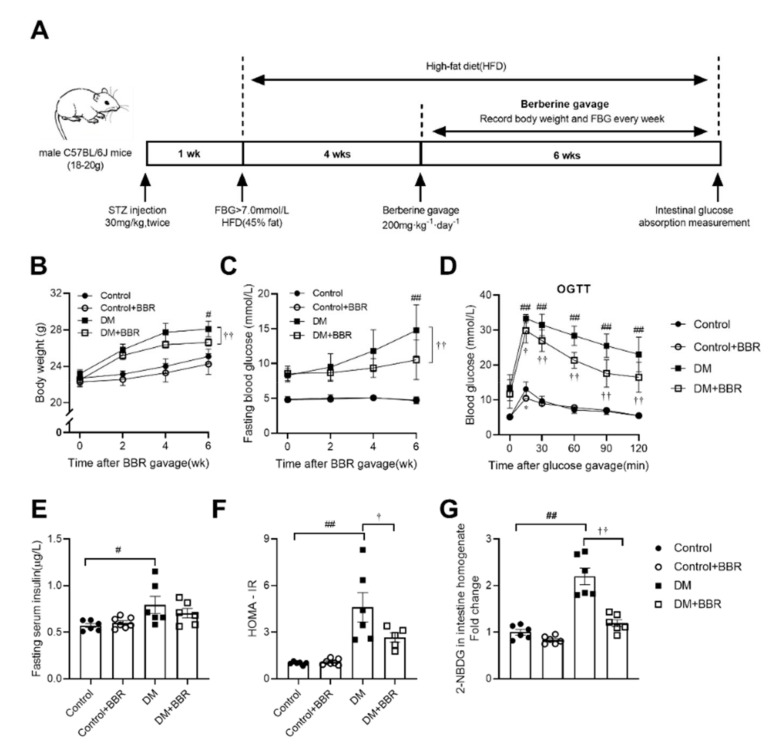
Berberine treatment (200 mg·kg^−1^·day^−1^, BW) for 6 weeks decreased body weight and fasting blood glucose and improved glucose tolerance and systemic insulin sensitivity and intestinal glucose absorption in diabetic mice. (**A**) Schematic representation of experimental protocol. (**B**,**C**) Body weight and fasting blood glucose changes in control, control + BBR, DM, and DM + BBR groups. (**D**) Berberine treatment for 6 weeks improved glucose tolerance in diabetic mice. (**E**,**F**) Berberine treatment for 6 weeks improved systemic insulin sensitivity in diabetic mice. (**G**) Berberine treatment for 6 weeks decreased intestinal 2-NBDG absorption in diabetic mice. Data are expressed as mean ± SEM (*n* = 6). Control, control mice; Control + BBR, control mice treated with berberine (200 mg·kg^−1^·day^−1^ gavage), DM, diabetic mice; DM + BBR, diabetic mice treated with berberine (200 mg·kg^−1^·day^−1^ gavage). Control vs. Control + BBR; Control vs. DM, # *p* < 0.05, ## *p* < 0.01; DM vs. DM + BBR, † *p* < 0.05, †† *p* < 0.01.

To examine the effects of berberine on blood glucose homeostasis in diabetic mice, body weight and fasting blood glucose were monitored, and serum insulin and HOMA-IR, which is a representative indicator for systemic insulin sensitivity, were tested. Body weight and fasting blood glucose were increased in mice in the DM group compared to mice in the control group (body weight, 28.08 ± 0.86 vs. 27.74 ± 0.33 g; fasting blood glucose, 14.77 ± 1.50 vs. 4.67 ± 0.18 mM, DM vs. Control, Figure 1B,C), while body weight and fasting blood glucose were decreased in the berberine treated diabetic mice compared to diabetic mice (body weight, 25.08 ± 0.81 vs. 28.08 ± 0.86 g; fasting blood glucose, 10.52 ± 1.27 vs. 14.77 ± 1.50 mM, DM + BBR vs. DM, Figure 1B,C). Mice in the DM group showed higher postprandial blood glucose than mice in the control group in OGTT (Figure 1D). After berberine treatment (200 mg·kg^−1^·day^−1^, BW) for 6 weeks, postprandial blood glucose was decreased in diabetic mice receiving berberine treatment compared to diabetic mice (Figure 1D), concomitant with improved systemic insulin sensitivity (Figure 1E,F). In order to evaluate the effect of berberine on intestinal glucose absorption directly, we observed the glucose transport capacity of intestine by 2-NBDG transport experiment ex vivo and found that ability of intestinal glucose absorption of diabetic mice was enhanced by 2.2 times compared to mice in the control group (Figure 1G). After berberine gavage treatment for 6 weeks, the ability of intestinal glucose absorption of mice in the DM + BBR group was decreased by 45.7% compared to the DM group (Figure 1G). Moreover, we found that glucose level in feces, which was normalized by food intake and body weight, was decreased by 30.4% in diabetic mice compared to mice in the control group and increased by 70.6% in mice in the DM + BBR group compared to mice in the DM group (Figure S1). Previous studies have shown high fat diet induced overgrowth of intestinal villus [19]. To examine berberine’s effect on intestinal epithelium, intestinal villus length and crypt depth were assessed using HE staining. After berberine treatment (200 mg·kg^−1^·day^−1^, BW) for 6 weeks, the intestinal crypt depth, but not villus length, was decreased in berberine treated diabetic mice (Figure S2). Besides beneficial effects of long-term administration of berberine on intestinal glucose absorption, we further tested instant effects by OGTT after single dose administration of berberine with glucose and found that a single dose gavage of berberine (200 mg·kg^−1^, BW) decreased postprandial blood glucose in OGTT in normal mice (Figure S3). 

### 2.2. Berberine Decreased GLUT2 Localization in BBM of Intestinal Epithelial Cells in Diabetic Mice

**Figure 2:**
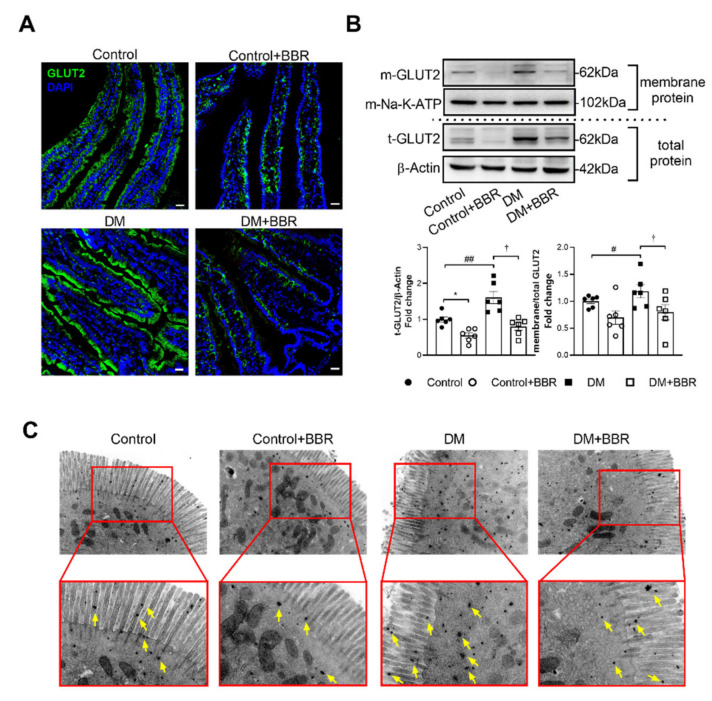
Berberine treatment decreased GLUT2 localization in BBM of intestinal epithelial cells in diabetic mice. (**A**) GLUT2 (green fluorescence) in BBM was decreased after berberine treatment (200 mg·kg^−1^·day^−1^, BW) for 6 weeks, bar = 20 μm, blue fluorescence (DAPI) represented for cell nucleus. (**B**) Berberine treatment (200 mg·kg^−1^·day^−1^, BW) for 6 weeks decreased intestinal GLUT2 expression and GLUT2 membrane localization. (**C**) Electron micrographs showed that GLUT2 positive gold particles on microvillus and the BBM were decreased in berberine treated diabetic mice (DM + BBR) (200 mg·kg^−1^·day^−1^, BW for 6 weeks), bar = 0.5 μm. Data are expressed as mean ± SEM (*n* = 6). m-Na-K-ATP was used as internal reference of the membrane. Control vs. Control + BBR, * *p* < 0.05; Control vs. DM, # *p* < 0.05, ## *p* < 0.01; DM vs. DM + BBR, † *p* < 0.05.

After berberine treatment (200 mg·kg^−1^·day^−1^, BW) for 6 weeks, expression of intestinal GLUT2 was decreased by 50.4% in mice in the DM + BBR group compared to mice in the DM group (Figure 2B). The subcellular distribution changes of GLUT2 in intestinal epithelial cells after berberine treatment were further studied, and we found that apical GLUT2 localization in brush border membrane of intestinal epithelial cells was increased in diabetic mice, as showed in membrane protein Western blotting (Figure 2B), immunofluorescence (Figure 2A) and immune electron microscopy tests (Figure 2C). After berberine treatment for 6 weeks, GLUT2 (shown as green fluorescence) in BBM was decreased in both control and diabetic mice after berberine treatment (Figure 2A). Furthermore, we observed berberine’s effects on GLUT2 distribution at subcellular level using the immunohistochemistry for electron microscopy; GLUT2 positive gold particles on microvillus were decreased in control and diabetic mice after berberine treatment (Figure 2C). SGLT1 is another glucose transporter in apical membrane of intestinal epithelial cells. We found that the expression of intestinal SGLT1 decreased by 38.3% in the DM + BBR group compared with the DM group (Figure S4).

### 2.3. Berberine Decreased Glucose Absorption by Inhibiting GLUT2 Translocation in IEC-6 Cells

**Figure 3:**
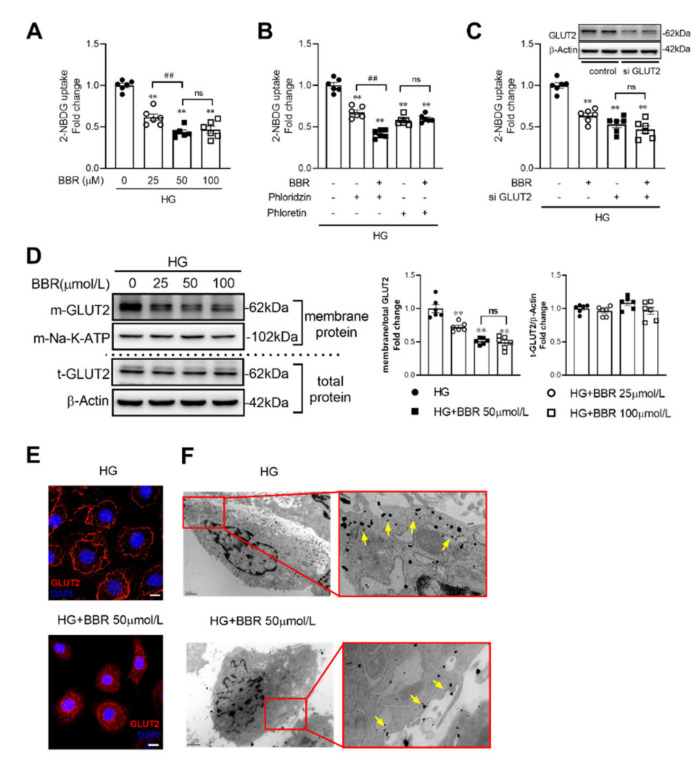
Berberine decreased glucose absorption in a dose-dependent manner through inhibiting GLUT2 translocation in IEC-6 cells. (**A**) Berberine decreased 2-NBDG uptake in a dose-dependent manner in IEC-6 cells. (**B**,**C**) phloretin, a GLUT2 inhibitor, rather than phloridzin, a SGLT1 inhibitor, blocked berberine’s effects on 2-NBDG uptake in IEC-6 cells. (**D**) siRNA for GLUT2 blocked berberine’s effects on cellular 2-NBDG uptake in IEC-6 cells. (**E**) GLUT2 (red fluorescence) in BBM was decreased berberine treated (50 μM) for 2 h, bar = 20 μm, blue fluorescence (DAPI) represented for cell nucleus. (**F**) Electron micrographs showed that GLUT2 positive gold particles at membrane were decreased in IEC-6 cells after berberine treatment (50 μM) for 2 h, bar = 1 μm under low power lens; bar = 400 nm under high power lens. HG, high concentration of glucose (50 mM); phloretin (0.5 mM), GLUT2 inhibitor; phloridzin (0.5 mM), SGLT1 inhibitor; si GLUT2, siRNA for GLUT2; Data are expressed as mean ± SEM (*n* = 6). m-Na-K-ATP was used as internal reference of the membrane. ** *p* < 0.01, vs. control; ## *p* < 0.01; ns, no significance.

A higher concentration of glucose (50 mM) was applied to culture IEC-6 cells to simulate high luminal concentration of glucose after food intake. We found that compared to control group, membrane localized GLUT2 was reduced by 27.7%, 48.9%, and 51.0% in the berberine 25, 50, and 100 μM groups, respectively (Figure 3A), Moreover, phloretin (0.5 mM), GLUT2 inhibitor, but not phloridzin (0.5 mM), one of SGLT1 inhibitors, could block the berberine’s effects of intestinal glucose absorption (Figure 2B). GLUT2 expression was knocked down by GLUT2 siRNA, which could also block cellular glucose uptake (Figure 2C). Immunofluorescence and western blot show that membrane localized GLUT2 of IEC-6 cells was decreased in berberine treatment group (Figure 3D,E). GLUT-2 positive gold particles on microvillus were decreased in HG + BBR group compared to HG group (Figure 3F).

### 2.4. Berberine Decreased GLUT2 Translocation and Glucose Uptake through Depressing PLC-β2 - GLUT2 Signal Pathway

**Figure 4:**
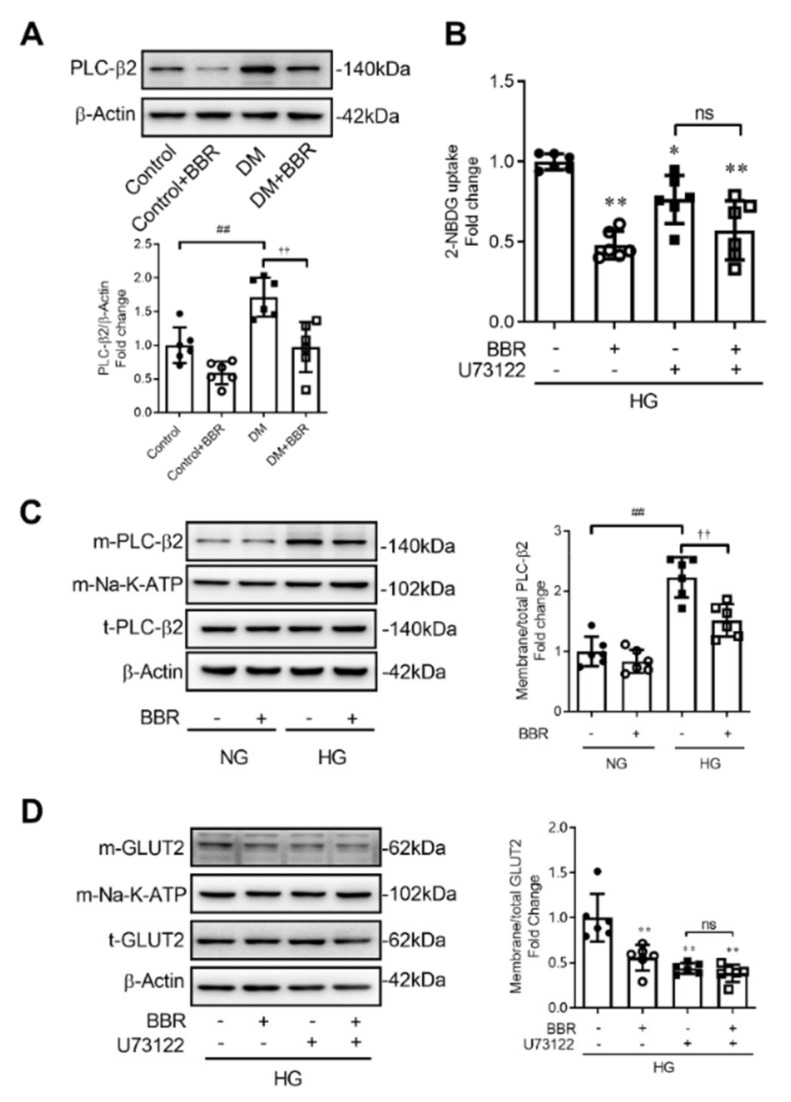
Berberine decreased GLUT2 translocation and glucose uptake through inhibiting the PLC-β2-GLUT2 signal pathway. (**A**) Berberine treatment (200 mg·kg^−1^·day^−1^, BW) for 6 weeks decreased intestinal PLC-β2 expression in the DM + BBR group compared to mice in the DM group. (**B**) U73122 (10 μM), one of the PLC-β2 inhibitors, blocked berberine’s effects on glucose uptake in IEC-6 cells. (**C**) Berberine decreased membrane localized PLC-β2 in IEC-6 cells. (**D**) U73122 (10 μM), one of the PLC-β2 inhibitors, blocked berberine’s effects on GLUT2 translocation. Data are expressed as mean ± SEM (*n* = 6). m-Na-K-ATP was used as internal reference of the membrane protein. HG, high concentration of glucose (50 mM); m-GLUT2, membrane localized GLUT2; t-GLUT2, total GLUT2; m-PLC-β2, membrane localized PLC-β2; t-PLC-β2, total PLC-β2; U73122, PLC-β2 inhibitor. * *p* < 0.05, ** *p* < 0.01, vs. control; ## *p* < 0.01, †† *p* < 0.01; ns, no significance.

Berberine treatment (200 mg·kg^−1^·day^−1^, BW) for 6 weeks decreased intestinal PLC-β2 expression by 43.3% in mice in the DM + BBR group compared to mice in the DM group (Figure 4A). To investigate the mechanism underlying berberine’s effect on the GLUT2 translocation, IEC-6 cells were cultured in high concentration of glucose (50 mM) to simulate intestinal glucose condition after food intake. We found that berberine (50 μM) treatment for 2 h decreased membrane localized PLC-β2 in IEC-6 cells (Figure 4C). U73122 (10 μM), one of PLC-β2 inhibitors, used to study the mechanism of berberine’s effect on GLUT2 translocation. Compared to the U73122 group, berberine treatment could not further reduce cellular glucose uptake and GLUT2 translocation in the U73122 + BBR group (Figure 4B,D), which suggested that U73122 blocked berberine’s effect on cellular glucose uptake and GLUT2 translocation. This evidence suggests that berberine decreases GLUT2 translocation and glucose uptake through inhibiting the PLC-β2-GLUT2 signal pathway.

### 2.5. Berberine Treatment for 6 Weeks Decreased Intestinal IGF-1, IGFBP-3 Levels, and IGF-1R Expression in Diabetic Mice

**Figure 5:**
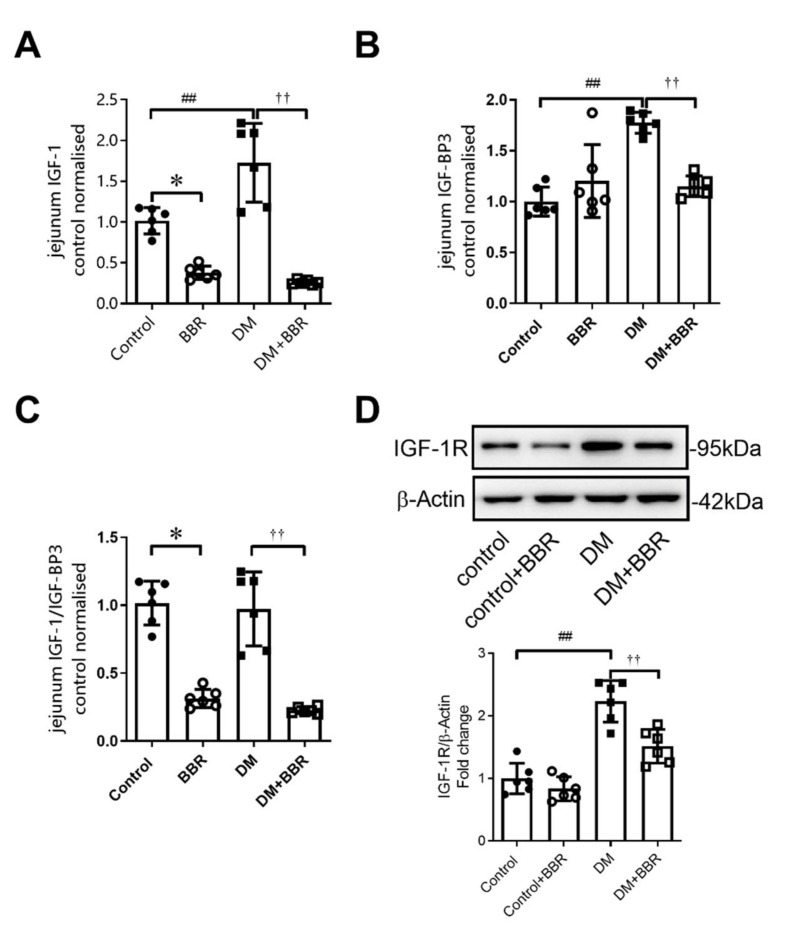
Berberine treatment (200 mg·kg^−1^·day^−1^, BW) for 6 weeks decreased intestinal IGF-1, IGFBP-3 levels, and IGF-1R expression in diabetic mice. (**A**–**C**) Intestinal IGF-1, IGFBP-3, and IGF-1/IGFBP-3 were decreased in diabetic mice after berberine treatment (200 mg·kg^−1^·day^−1^, BW) for 6 weeks. (**D**) Intestinal IGF-1R expression were decreased in diabetic mice after berberine treatment (200 mg·kg^−1^·day^−1^, BW) for 6 weeks. Data are expressed as mean ± SEM (*n* = 6). Control, control mice; Control + BBR, control mice treated with berberine (200 mg·kg^−1^·day^−1^ gavage), DM, diabetic mice; DM + BBR, diabetic mice treated with berberine (200 mg·kg^−1^·day^−1^ gavage). Control vs. Control + BBR, * *p* < 0.05; Control vs. DM, ## *p* < 0.01; DM vs. DM + BBR, †† *p* < 0.01.

IGF-1, IGFBP, and IGF-1R have been considered as important regulators in glucose metabolic homeostasis [20,21]. In our study, intestinal IGF-1 and IGFBP-3 were elevated by 72.7% and 77.4% in diabetic mice compared to control (Figure 5A–C), and berberine treatment decreased intestinal IGF-1, IGFBP-3, and IGF-1/IGFBP-3 level by84.8%, 35.2%, and 76.6% (Figure 5A–C). Intestinal IGF-1R expression was also increased by 2.23 times in diabetic mice (Figure 5D), and berberine treatment decreased intestinal IGF-1R expression by 32.1% (Figure 5D). 

### 2.6. Berberine Decreased GLUT2 Translocation and Glucose Uptake through Inhibiting IGF-1R-PLC-β2-GLUT2 Signal Pathway

**Figure 6:**
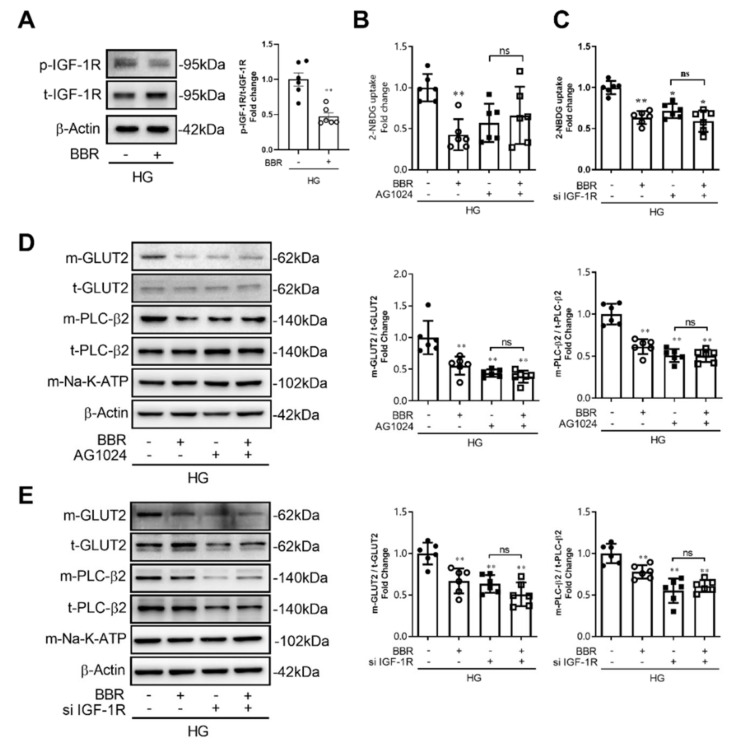
Berberine decreased GLUT2 translocation and glucose uptake through inhibiting the IGF-1R-PLC-β2-GLUT2 signal pathway. (**A**) Berberine decreased phosphorylation of IGF-1R in IEC-6 cells. (**B**,**C**) AG1024 (10 μM) and siRNA for IGF-1R blocked berberine’s effects on 2-NBDG uptake in IEC-6 cells. (**D**,**E**) AG1024 (10 μM) and siRNA for IGF-1R blocked berberine’s effects on GLUT2 translocation and PLC-β2 membrane localization in IEC-6 cells. Data are expressed as mean ± SEM (*n* = 6). HG, high concentration of glucose (50 mM); m-GLUT2, membrane localized GLUT2; t-GLUT2, total GLUT2; m-PLC-β2, membrane localized PLC-β2; t- PLC-β2, total PLC-β2; p-IGF-1R, phosphorylation of IGF-1R; t-IGF-1R, total IGF-1R. AG1024, IGF-1R inhibitor. si IGF-1R, siRNA for IGF-1R. * *p* < 0.05, ** *p* < 0.01, vs. control; ns, no significance.

To investigate the roles of intestinal IGF-1R in PLC-β2 mediated GLUT2 translocation and glucose absorption, AG1024 (10 μM), one of IGF-1R inhibitors, and knocking down of IGF-1R by siRNA were applied. We found that berberine (50 μM) for 2 h decreased phosphorylation of IGF-1R in IEC-6 cells (Figure 6A). AG1024 blocked the berberine’s effect on cellular glucose uptake (Figure 6B). Then, as shown in Figure 6D, compared to AG1024 group, berberine treatment could not further reduce membrane localized GLUT2 in IEC-6 cells in AG1024 + BBR group, and AG1024 also blocked berberine’s effect on PLC-β2 membrane localization, which suggested that IGF-1R is the upstream mechanism that regulates PLC-β2 localization. Moreover, we knocked down the expression of IGF-1R in IEC-6 cells by siRNA, and found that berberine’s effects on cellular glucose uptake could be blocked by knocking down the expression of IGF-1R (Figure 6C); GLUT2 translocation (Figure 6E) and PLC-β2 localization in membrane (Figure 6E) were also blocked by siRNA for IGF-1R. This evidence suggests that berberine decreases GLUT2 translocation and glucose uptake through inhibiting the IGF-1R-PLC-β2-GLUT2 signal pathway.

## 3. Discussion

This study investigated the effects of berberine on intestinal glucose absorption. The results showed that berberine reduced intestinal glucose absorption by decreasing localization of GLUT2 in the brush border membrane of intestinal epithelial cells, through inhibiting IGF-1R-PLC-β2-GLUT2 pathway. These findings indicate that berberine has potential as an adjunctive medication for the management of enhanced intestinal glucose absorption and postprandial hyperglycemia in diabetes. 

Postprandial hyperglycemia is the most common symptom in type 2 diabetes mellitus and also an important pathogenic factor and independent risk factor of many diabetic complications [22,23]. It is suggested that pathological enhancement of intestinal glucose absorption plays pivotal roles in postprandial hyperglycemia [1,24]. In the present study, we found that intestinal glucose absorption was enhanced in diabetic conditions, which was mainly evidenced by the increased ability of intestinal glucose transport in vitro and permanent localization of GLUT2 in the BBM of intestinal epithelium in diabetic mice. At the same time, body weight, fasting blood glucose, and postprandial blood glucose showed abnormal changes in diabetic mice. There are several medical approaches for postprandial hyperglycemia, including stimulating insulin secretion, improving insulin sensitivity to increase glucose uptake by muscle and adipose tissue, and inhibiting glucose reabsorption in the kidney. Given the dominant role of the intestine as source of blood glucose, inhibiting intestinal sugar digestion progress becomes a common approach to control postprandial hyperglycemia; however, there were few drugs targeting intestinal glucose absorption.

Berberine, an isoquinoline alkaloid from Rhizoma coptidis, is a traditional oriental medicine found to be effective in the treatment of cancer, inflammation, and metabolic diseases, especially type 2 diabetes mellitus [25]. Our previous studies reported that berberine could reduce ischemia/reperfusion-induced myocardial apoptosis, improve mesenteric artery insulin sensitivity in diabetes [26], and reduce diabetic myocardial damage by inhibiting IGF-1R signaling [13]. However, low bioavailability and an unidentified pharmacological mechanism are the main barriers to clinical application of berberine [27]. In addition to its pharmacological anti-diabetic roles in peripheral organs, it has been shown that berberine inhibits the function of disaccharide, promotes the release of GLP-1 to regulate metabolism, and modulates the component part of intestinal microbiota [28]. However, whether berberine could ameliorate intestinal glucose absorption received little attention. 

Consistent with previous studies, we found that berberine treatment for six weeks could decrease body weight and fasting blood glucose in diabetic mice and improve glucose tolerance and systemic insulin sensitivity. Berberine’s beneficial effects on body weight are attributed to multiple mechanisms, such as inhibiting lipogenesis, decreasing food intake, and stimulating energy expenditure, while the blood glucose lowering effects also contribute to reducing body weight [25]. The present study aimed to explore berberine’s effects on intestinal glucose absorption, the main source of blood glucose, in T2DM. Using the method of 2-NBDG uptake, we found that berberine decreased intestinal glucose transport, which indicated that berberine decreased intestinal glucose absorption in diabetes. Mechanistically, berberine decreased the ability of cellular glucose uptake of IEC-6 cells in a dose dependent manner. The results suggest that in addition to direct effects of lowing blood glucose, berberine exerts indirect beneficial effect by inhibiting intestinal glucose absorption. We found the feces glucose level was increased after berberine treatment for 6 weeks, which might facilitate the genesis of short chain fatty acid in the large intestine [29], and the resultant lower blood glucose could improve tight junctions between intestinal epithelial cells and reduce damage caused by inflammatory factors [30]. We did not assess the instant effect of single dose of berberine on diabetic intestinal blood glucose, especially the time of duration of inhibiting effect, which became one of the limitations of current study. However, we implemented OGTT in normal mice with a single dose berberine treatment, and found the same postprandial blood glucose lowering effect, which verified the immediate effect of berberine observed in IEC-6 cells.

SGLT1 and GLUT2 are major intestinal glucose transporters [31]. SGLT1 plays the dominant role in glucose absorption when glucose concentration is less than 30 mM, while GLUT2 makes the main contribution when glucose concentration is above 30 mM after food intake [3]. Translocation of GLUT2 from cytoplasm to BBM mediates massive glucose absorption, while internalization of GLUT2 from BBM to cytoplasm prevents postprandial hyperglycemia, which constitutes a dynamic balance [32]. However, permanent localization of GLUT2 in BBM occurs in diabetic condition [5], which is closely related to postprandial hyperglycemia. Our study found that GLUT2 inhibitor phloretin, but not SGLT1 inhibitor phloridzin, could block berberine’s effects of decreasing cellular glucose uptake under the condition of high concentration of glucose, which indicated that GLUT2 was the important target transporter that mediates berberine’s effects of reducing intestinal glucose absorption. Nonetheless, we cannot rule out the possibility that berberine’s effects of suppressing SGLT1 also contribute to decreasing blood glucose, especially during long term administration. Given the fact that GLUT2-mediated glucose absorption is the predominant part under postprandial condition, we examined berberine’s effects on GLUT2 expression and translocation and found that berberine reduced localization of GLUT2 in the BBM of intestinal epithelium. A similar reduction of GLUT2 localization in berberine treated IEC-6 cells was observed under the condition of high concentration of glucose (50 mM), which was designed to simulate luminal glucose level after food intake. The translocation of GLUT2 relies on the regulation of various signal molecules. As is described in previous studies, G protein effector PLC-β2 mediates over 70% of GLUT2 translocation during stimulation of high concentration of glucose, which is the vital factor facilitating GLUT2 translocation to BBM [33]. In the present study, we found that berberine decreased the expression of PLC-β2 in diabetic mice and membrane-located PLC-β2 in IEC-6 cells. Additionally, U73122, an inhibitor of PLC-β2, blocked the effects of berberine of decreasing cellular glucose uptake and GLUT2 translocation. These data suggest that PLC-β2 mediated berberine’s effects on cellular glucose uptake. 

IGF-1/IGF-BP plays important roles in metabolic homeostasis, and the specific functions of the IGF-1 receptors on glucose transporter proteins have been studied in several cell types and organs [34,35,36,37]. However, the role of IGF-1R in intestine, especially the possible relationship between IGF-1R and intestinal GLUT2, was unclear. In the present study, IGF-1 and IGFBP-3 level was increased in diabetic intestinal tissues, and the expression of IGF-1R was also elevated, which was reversed after berberine gavage treatment for 6 weeks. However, whether IGF-1R changes mediated berberine’s effects on intestinal glucose absorption and intestinal GLUT2 translocation is still an open question. Consistent with IGF-1R changes in intestinal tissues, berberine down-regulated phosphorylation of IGF-1R in IEC-6 cells. Furthermore, by using AG1024, an inhibitor of IGF-1R and siRNA knocking down technique, we found that inhibiting IGF-1R could block berberine’s effects of reducing GLUT2 translocation and cellular glucose uptake in IEC-6 cells. In addition, berberine decreasing membrane localization of PLC-β2 could also be blocked by inhibiting IGF-1R. Taken together, berberine decreased GLUT2 translocation and cellular glucose uptake in IEC-6 cells by inhibiting IGF-1R-PLC-β2-GLUT2 pathway. Further studies are warranted to delineate berberine effects on the interaction between IGF-1R and PLC-β2. Several studies have indicated that tyrosine kinase IGF-1R did have a regulatory effect on G protein coupled receptor mediated by PLC signaling [38,39]. The physiological functions of this regulatory network in glucose homeostasis need more research in the future.

## 4. Materials and Methods

### 4.1. Animals

C57BL/6J mice weighting 18–20 g were from the Animal Center of the Air Force Military Medical University, Xi’an, China. The animals were housed in a temperature-controlled room (25–28 °C) with a 12 h light dark cycle. Mice were fed a standard diet with free access to d-water. In order to induce hyperglycemia condition in a short time, type 2 diabetic mice were induced by low dose injection of STZ (Sigma-Aldrich, Shanghai, China) combined with a high fat diet (Medicine Ltd, Jiangsu, China) as previously described [26,40]. In brief (Figure 1A), after 1 week of adaptation, mice were made to fast overnight and were then injected (i.p.) with STZ (streptozotocin, 30 mg·kg^−1^, BW), which was dissolved in citrate buffer (pH 4.5) to induce diabetes, and a second injection was applied 3-days later. Normal control animals (Control) were made to fast overnight and injected with the citrate buffer vehicle and fed a standard diet (containing 22% calories from fat. MD17121, Medicine Ltd, Jiangsu, China). Seven days later, the fasting blood glucose of STZ-treated mice was measured; mice with fasting blood glucose levels over 7.0 mM were randomly divided into two groups and continued to be fed the high-fat diet (containing 45% calories from fat. MD12032, Medicine Ltd, Jiangsu, China) for 4 weeks. These two groups were matched for body weight and blood glucose levels. One group was used as the diabetic control (DM), and the other (DM + BBR) was treated with berberine chloride (J&K Scientific Ltd, Beijing, China) dissolved in 0.9% saline solution at a dose of 200 mg·kg^−1^·day^−1^; the same berberine administration program was given to the berberine treated normal group (Control + BBR). The normal control and diabetic control mice were treated with 0.9% saline solution (gavage). During the process of berberine treatment, body weight and fasting blood glucose were monitored. At the end of the study, animals were made to fast overnight (10 h) and killed by administration of an overdose of anesthetic, sodium-pentobarbital (30 mg·kg^−1^, i.p.). All experimental procedures were performed in accordance with International Guidelines for the Care and Use of Laboratory Animals and were approved by the Animal Care and Research Ethics Committee of the Air Force Military Medical University Research Council. 

### 4.2. Glucose Tolerance Test

After berberine treatment (200 mg·kg^−1^·day^−1^) for 6 weeks, mice from 4 groups (Control, Control + BBR, DM, DM + BBR) were made to fast overnight (10 h) and gavage administered with D-glucose (2 g·kg^−1^, BW). The blood was taken at 0, 15, 30, 60, and 120 min after oral glucose administration, and blood glucose levels (milligram per deciliter) were determined (ACCU-CHEK; Roche Diagnostic, Meylan, France). These experiments were performed at least with 6 individual animals.

### 4.3. Intestine Glucose Transport Test and Cellular Glucose Uptake Test

Intestinal glucose transport test was performed using jejunal sacs from 4 groups (Control, Control + BBR, DM, DM + BBR) of mice. Animals were made to fast for 10 h and were killed by overdose injection of pentobarbital (30 mg·kg^−1^, i.p.). The proximal intestinal was dissected and rinsed in cold saline solution. Jejunal loops (3 cms long) were prepared and 0.5 mL of KRB solution (MgCl_2_ 0.49 mM, NaCl 119.78 mM, KCl 4.56 mM, Na_2_HPO_4_ 0.70 mM, NaH_2_PO_4_ 1.30 mM, NaHCO₃ 14.99 mM) with 50 mM glucose and 10 nM 2-NBDG (sigma, USA) was incubated inside the jejunal lumen for 30 min in 3 mL oxygenized KRB solution at 37 °C. The fluorescence intensity was measured in intestinal tissue protein lysate by using FLUOstar Omega Micro plate Reader (BMG LABTECH) and used to calculate 2-NBDG transport as concentration standardized fluorescence intensity [41]. At least 6 independent experiments were performed.

Cellular glucose uptake test was performed using rat intestinal epithelial cells line IEC-6, which was obtained from Jennio Biotech Co. Ltd (Guangzhou, China) at passage 3. Berberine was dissolved in DMSO to make a stock solution and added to culture medium to dilute to target concentration. The concentration of DMSO was kept below 0.1% in all the cell cultures and did not exert any detectable effect on cell growth or death. After berberine treatment according to specific experimental purpose, IEC-6 cells were harvested in the glucose-free DMEM and then incubated with 10 nM of 2-NBDG at 37 °C for 30 min. The uptake reaction was stopped by washing the cells 3 times with Phosphate-buffered saline, pH 7.4. The intensity of 2-NBDG fluorescence of the cell’s lysate was measured using FLUOstar Omega Micro plate Reader. Protein concentration of the cell lysate was measured, and the ratio of fluorescence intensity to protein concentration was an indicator of cellular glucose uptake [42].

### 4.4. Immunofluorescence Histochemistry and Immunohistochemistry for Electron Microscopy

Immunofluorescence was performed on intestines, which were stained with GLUT2 antibody (from Santa Cruz, SC-9117, 1:100, Dallas, TX, USA) and counterstained with DAPI. As for immunohistochemistry for electron microscopy, the intestine tissues and IEC-6 cells from each group were fixed with 4% paraformaldehyde containing 0.5% glutaraldehyde for 4 h, and then, intestinal sections and cells were immersed in PBS containing 5% BSA and 0.05% Triton X-100 for 4 h to block nonspecific immunoreactivity. Samples were incubated with rabbit anti-GLUT2 (Santa Cruz, SC-9117, 1:100, Dallas, TX, USA) for over 24 h and then were incubated with goat anti-rabbit IgG conjugated to 1.4 nm gold grains (1:100, Nano probes, Stony Brook, NY, USA) overnight. Silver enhancement was carried out in the dark with the HQ Silver Kit (Nano probes) for visualization of GLUT2 immunoreactivity. Before and after the silver enhancement step, samples were rinsed several times with deionized water. Immunolabeled samples were fixed with 0.5% osmium tetroxide in 0.1 M phosphate buffer for 2 h, dehydrated in graded ethanol series, then in propylene oxide, and finally flat-embedded in Epon 812. After polymerization, samples were trimmed under a stereomicroscope and mounted onto blank resin stubs. Ultrathin sections were cut with an ultratome (Nova, LKB, Bromma, Sweden) and mounted on mesh grids (six to eight sections/grid). The ultrathin sections were counterstained with uranylace-tate and lead citrate and observed under a JEM-1230 electron microscope (JEOL Ltd., Tokyo, Japan) [43].

### 4.5. Western Blot Analysis

Intestinal tissue and IEC-6 cells membrane protein was isolated by MinuteTM Plasma Membrane Protein Isolation Kit (Invent Biotechnologies, Inc. Plymouth, MN, USA) [44]. Total proteins of intestinal tissue and IEC-6 cells were extracted in lysis buffer (25 mM Tris-HCl pH7.4, 150 mM NaCl, 1% NP-40, 1 mM EDTA, 5% glycerol, 0.1% SDS) containing protease and phosphatase inhibitors. Then, 20 μg of protein were subjected to SDS-PAGE and transferred to PVDF membranes for immune-blot analysis. The following antibodies were used: GLUT2 (Santa Cruz, SC-9117, 1:500, Dallas, TX, USA), GLUT5 (Santa Cruz, SC-30109, 1:500, Dallas, TX, USA), SGLT1 (Abcam, ab14686, 1:500, Waltham, MA, USA), PLC-β2 (Santa Cruz, SC-206, 1:500, Dallas, TX, USA), IGF-1R-β (Cell Signaling Technologies, 9750S, 1:1000, Shanghai, China), phospho-IGF-1R-β (Tyr 1135) (Cell Signaling Technologies, 3918, 1:1000, Shanghai, China), β-Actin (Cell Signaling Technologies, 3700S, 1:1000, Shanghai, China), Anti-ATP1A2 (Abcam, ab2871, 1:1000, Waltham, MA, USA). The intensity of the specific immune-reactive bands was quantified using Quantity One software (Bio-Rad Laboratories, Inc. Hercules, CA, USA).

### 4.6. Measurement of Hormone

Fresh serum from each group mice was separated from whole blood by centrifugation at 5000× *g* for 10 min at 4 °C after the formation of a clot in a blood collection tube and stored at 4 °C. Intestine tissue lysate is used to test hormone level after standardization of protein concentration among all groups [45]. Serum concentration of insulin was tested using ELISA assays (ExCell Bio Co., Ltd. Jiangsu, China). Serum concentration and intestine hormone levels of IGF-1 and IGFBP-3 were tested using ELISA assays (4A Biotech Co., Ltd. Beijing, China). Each hormone was tested at least with 6 individual animals.

### 4.7. Small Interfering RNA Design and Transfection

The cognate siRNA against GLUT2 and IGF-1R was designed and purchased (Genepharm, Shanghai, China) along with a scrambled control siRNA. IEC-6 cells were seeded and transfected with siRNA to a final concentration of 20 nM using Lipofectamine™ RNAiMAX Transfection Reagent (Thermo Fisher Scientific, Shanghai, China) when cells reached 30–50% confluence. Protein knockdown efficiencies were assessed at 72 h after transfection. The siRNA sequences used in this study were as follows: GLUT-2, sense 5′-GCACCUCAAGAGGUAAUAA-3′, anti-sense 5′-UUAUUACCUCUUGAGGUGC-3′; IGF-1R, sense 5′-GCCGACACUACUACUACAATT-3′, anti-sense 5′-UUGUAGUAGUAGUGUCGGCTT-3′; non-functional mutant, sense 5′-AAGAGAAAAAGCGAAGAGCCA-3′, anti-sense 5′-UGGCUCUUCGCUUUUUCUCUU-3′.

### 4.8. Feces Glucose Test

The normal diet was given to all groups of mice at 72 h prior to feces glucose test to exclude any diet-specific effects on feces glucose levels. Fresh padding and the same quantity of feed were given to all groups of mice; the residual feed in each group and body weight of mice were weighed after 24 h, and 20 grains of mice feces were collected in each group. Same protocol was repeated another 5 times to collect data of food intake and the feces sample, drying the feces by drying box (60 °C, 60 min) and pulverizing and thoroughly mixing them. The same volume of d-water was added to same weight of feces, and the suspension liquid was vibrated for 5 min and then centrifuged 1000× *g* for 10 min. The supernatant was reserved and centrifuged 2000× *g* for 10 min. The final supernatant was reserved as the sample to run the glucose level test by using a glucose determination Kit (rsbio Ltd. Shanghai, China). Glucose levels in feces were then normalized by food intake and body weight.

### 4.9. Statistical Analysis

All values are presented as mean ± SEM. Dunnett-t test was used to evaluate the statistical significance of difference between experimental and control groups, while SNK-q test was used between different experimental groups. Probabilities of <0.05 were considered to be statistically significant. All of the statistical tests were performed with the Graphpad Prism software, version 8.0 (Graph Pad Software, San Diego, CA, USA). The data and statistical analysis comply with the recommendations on experimental design and analysis in pharmacology.

## 5. Conclusions

**Figure 7:**
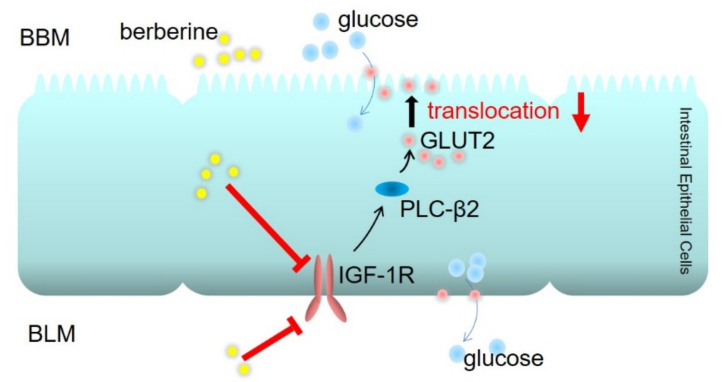
Schematic graph that illustrates the mechanisms of berberine for depressing intestinal glucose absorption and decreasing postprandial hyperglycemia. Through the inhibition of the IGF-1R-PLC-β2-GLUT2 signal pathway, berberine decreases intestinal glucose absorption and improves postprandial hyperglycemia in diabetic mice.

In summary, berberine reduced intestinal glucose absorption through decreasing localization of GLUT2 in the brush border membrane of intestinal epithelial cells, which might be the major mechanism of berberine for improving postprandial blood glucose in T2DM. The underlying mechanism of berberine for regulating the translocation of GLUT2 is through the inhibition of IGF-1R-PLC-β2-GLUT2 signaling pathway (Figure 7). These findings suggest berberine as potential adjunctive medication for the management postprandial hyperglycemia in diabetes.

## Data Availability

Not applicable.

## Supplementary Materials

The following are available online at https://www.mdpi.com/article/10.3390/ijms23010327/s1.
