# Supplementary material for: Berberine Decreases Intestinal GLUT2 Translocation and Reduces Intestinal Glucose Absorption in Mice
Source: Int J Mol Sci. 2021 Dec 28;23(1):327. doi: 10.3390/ijms23010327 (PMC8745600; doi:10.3390/ijms23010327)
Supplement: Supplementary file 1 [file ijms-23-00327-s001.zip › ijms-1473114-supplementary.pdf]

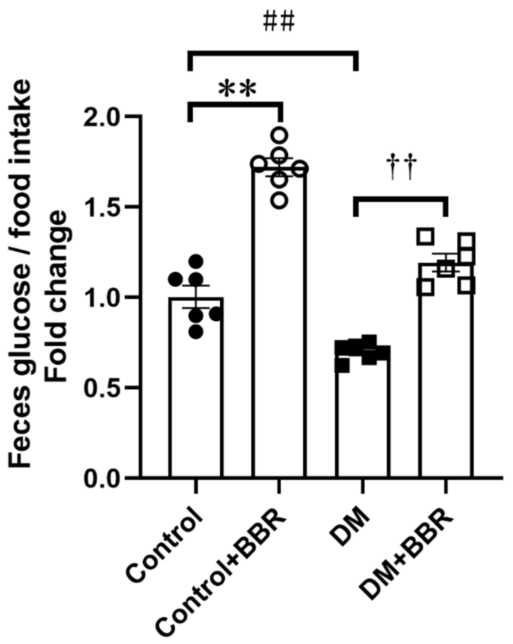

Figure S1. Berberine treatment (200 mg•kg-1•day-1, BW) for 6 weeks decreased feces glucose in diabetic mice. Data are expressed as mean ± SEM (n=6). Control, control mice; Control + BBR, control mice treated with berberine (200 mg•kg-1•day-1 gavage), DM, diabetic mice; DM + BBR, diabetic mice treated with berberine (200 mg•kg-1•day-1 gavage). Control vs. Control + BBR, * $p < 0.05$, ** $p < 0.01$; Control vs. DM, # $p < 0.05$, ## $p < 0.01$; DM vs. DM + BBR, † $p < 0.05$, †† $p < 0.01$.

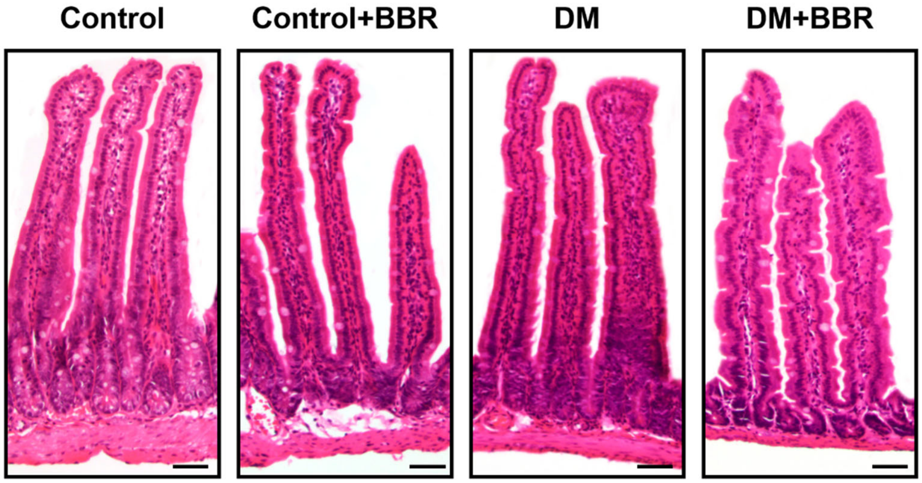

Figure S2. Berberine treatment (200 mg•kg-1•day-1, BW) for 6 weeks decreased intestinal crypt depth but not villus length in diabetic mice. Representative H&E staining of small intestine of each group. Control, control mice; Control + BBR, control mice treated with berberine (200 mg•kg-1•day-1 gavage), DM, diabetic mice; DM + BBR, diabetic mice treated with berberine (200 mg•kg-1•day-1 gavage). Scale bar: 100 μm.

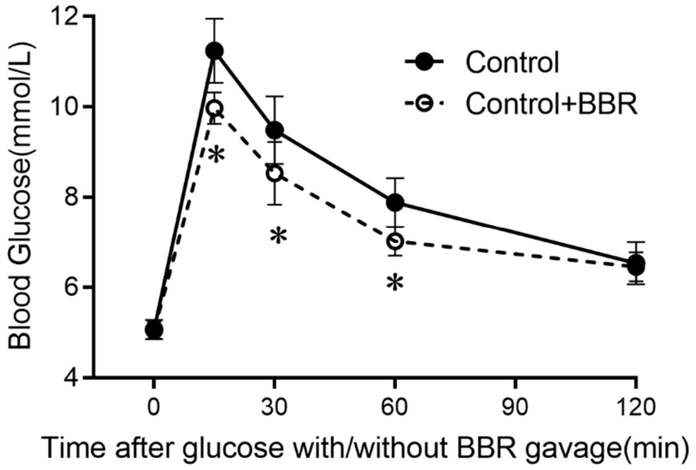

Figure S3. A single dose gavage of berberine (200 mg•kg-1, BW) decreased postprandial blood glucose in normal mice. Data are expressed as mean ± SEM (n=6). Control, normal mice garaged by glucose; Control + BBR, control mice treated with a single dose gavage of berberine (200 mg•kg-1, BW) +glucose, Control vs. Control + BBR, * $p< 0.05$

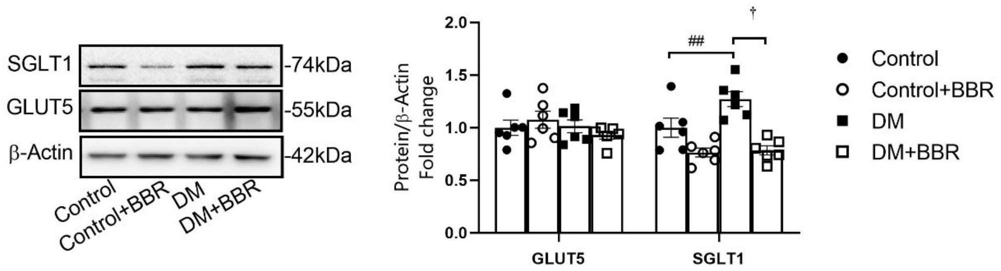

Figure S4. Berberine treatment (200 mg•kg-1•day-1, BW) for 6 weeks decreased intestinal SGLT1 expression, but not GLUT5, in diabetic mice. Data are expressed as mean ± SEM (n=6). Control, control mice; Control + BBR, control mice treated with berberine (200 mg•kg-1•day-1 gavage), DM, diabetic mice; DM + BBR, diabetic mice treated with berberine (200 mg•kg-1•day-1 gavage). Control vs. DM, # $p < 0.05$, ## $p < 0.01$; DM vs. DM + BBR, † $p < 0.05$, †† $p < 0.01$.
